# Prevention of Cholestatic Liver Disease Through BCL6-FXR Enterohepatic Crosstalk

**DOI:** 10.1016/j.jcmgh.2025.101706

**Published:** 2025-12-11

**Authors:** Ellen Fruzyna, Meredith A. Sommars, Yasu Omura, Kristine M. Yarnoff, Janice C. Wang, Christopher R. Futtner, Richard M. Green, Grant D. Barish

**Affiliations:** 1 Division of Endocrinology, Metabolism, and Molecular Medicine, Department of Medicine, Feinberg School of Medicine, Northwestern University, Chicago, Illinois; 2 Department of Biological and Health Sciences, Wheaton College, Wheaton, Illinois; 3 Jesse Brown VA Medical Center, Chicago, Illinois; 4 Division of Gastroenterology and Hepatology, Department of Medicine, Feinberg School of Medicine, Northwestern University, Chicago, Illinois

**Keywords:** BCL6, Bile Acid, Cholestasis, FXR, Liver

## Abstract

**Background & Aims:**

Bile acid (BA) metabolism must be tightly regulated because BAs serve as metabolic signaling molecules but become cytotoxic at high levels. The farnesoid X receptor (FXR) is a crucial BA sensor, yet our understanding of its regulation and coordination with other transcription factors is limited. Here, we investigated the role of B-cell lymphoma 6 (BCL6) in regulating BA levels and how it coordinates with FXR to protect from BA overload.

**Methods:**

We quantified cholesterol, BA levels, expression of key BA regulators, and hepatic damage markers in genetic mouse models with hepatic deletion of *Bcl6* (*Bcl6*^*LKO*^), global deletion of *Fxr* (*Fxr*^*KO*^), or combined loss of both factors.

**Results:**

We identified an epigenomic link between BCL6- and FXR-regulated gene networks. BCL6 regulated BA homeostasis through multiple mechanisms, including suppression of BA synthesis, activation of fibroblast growth factor receptor 4 (FGFR4) expression to sensitize hepatocytes to FGF15-mediated repression of *Cyp7a1*, and induction of the BA reuptake transporter sodium taurocholate cotransporting polypeptide (NTCP). Combined loss of hepatic *Bcl6* and whole body *Fxr* resulted in severe BA accumulation and hepatotoxicity, driven by a near-complete loss of hepatic small heterodimer partner (*Shp*), indicating that BCL6 and FXR co-repress BA synthesis and maintain BA homeostasis.

**Conclusions:**

These findings identify BCL6 as a previously unrecognized integrator of FXR-mediated enterohepatic signaling and a critical regulator of BA metabolism, acting through both FXR-dependent and FXR-independent mechanisms to maintain BA homeostasis and protect the liver from BA-induced injury.

## Graphical abstract

**Figure:**
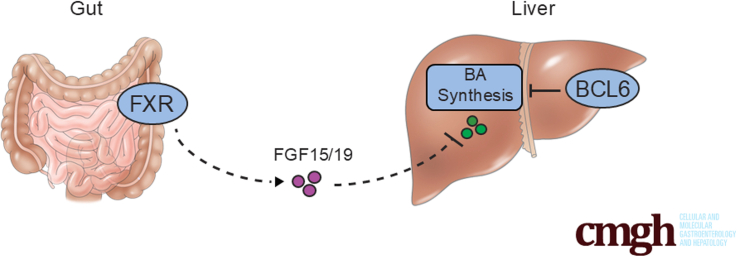

**Summary**

This study shows that hepatic B-cell lymphoma 6 controls bile acid metabolism and converges with farnesoid X receptor enterohepatic signaling. Combined loss of hepatic B-cell lymphoma 6 and farnesoid X receptor causes severe cholestatic liver disease.

**What You Need to Know**

**Background**

The transcriptional repressor B cell lymphoma 6 (BCL6) is a well-established regulator of immune cell function, but its role in metabolic tissues is less understood.

**Impact**

Here, we find that hepatic BCL6 cooperates with farnesoid X receptor to inhibit bile acid (BA) synthesis and levels, particularly in male mice.

**Future Directions**

Future studies should extend our findings to determine the impact of hepatic BCL6 BA regulation on intestinal BAs, microbiota, and immunity.

The liver orchestrates the body’s response to changing energy conditions by directing gene expression and metabolism in response to nutrient-induced cues. Among these signals are bile acids (BAs), which are released into the intestines following meals to aid in lipid digestion.^1^^,^^2^ BAs signal by activating farnesoid X receptor (FXR) in the liver to direct transcription controlling BA, carbohydrate, and lipid homeostasis.^2^, ^3^, ^4^, ^5^ Additionally, intestinal bile acids activate FXR in enterocytes and upregulate the production and release of fibroblast growth factor (FGF)19 (FGF15 in mice).^6^ FGF15/19 then circulates back to hepatocytes to shut off BA synthesis by inhibiting expression of the rate-limiting BA synthetic enzyme, *Cyp7a1*.^7^^,^^8^ Hepatic FXR can also bind to BAs and inhibit *Cyp7a1*, although this liver-intrinsic pathway is less critical.^9^, ^10^, ^11^, ^12^ In this manner, FXR senses high enterohepatic BA levels and communicates to the liver to reduce BA synthesis. FXR also induces liver BA removal by activating the BA efflux pump bile salt export pump (BSEP) and inhibiting the BA re-uptake transporter sodium-taurocholate cotransporting polypeptide (NTCP).^13^, ^14^

When BA homeostasis is disrupted, BAs can overload the liver and cause damage due to their detergent properties.^15^^,^^16^ Based on the role for FXR in repressing BA synthesis and enhancing BA efflux, FXR agonists have been developed as clinical therapeutics for cholestatic liver diseases. Although they are effective, they have drawbacks including incomplete clinical response,^17^ as well as a range of side effects such as pruritus and increased low-density lipoprotein (LDL) cholesterol levels.^18^ Thus, additional strategies to treat cholestatic diseases are needed.

Previously, our laboratory identified the transcriptional repressor B-cell lymphoma 6 (BCL6)^19^, ^20^, ^21^ as a negative regulator of fasting hepatic lipid metabolism and antagonist of peroxisome proliferator-activated receptor alpha (PPARa)-driven gene regulation.^22^ In addition to its role in fatty acid handling, prior work has linked hepatic PPARa to the control of enzymes and transporters of BAs,^23^ whereas studies in human hepatocytes demonstrate that the *PPARa* promoter contains an FXR binding site, and *PPARa* expression is induced by BAs.^24^ Together, these suggested that BCL6 and FXR control overlapping transcriptional pathways potentially involved in BA signaling. Furthermore, a link between BCL6 and circulating lipoprotein cholesterol has been described, indicating a role in sterol metabolism.^25^

Here, using a machine learning-based analysis of BCL6-controlled genes and active DNA regulatory regions in liver, we identified an epigenomic link between BCL6- and FXR-regulated gene networks. These results, together with the known roles for BCL6 and FXR in directing fed state liver metabolism,^22^^,^^26^ prompted us to examine whether BCL6 influences hepatic FXR signaling and BAs. We discovered that BCL6 controls BAs by: (1) suppressing BA synthesis; (2) activating expression of fibroblast growth factor receptor 4 (FGFR4), priming the liver for FGF15-mediated *Cyp7a1* repression; and (3) increasing expression of the BA re-uptake transporter NTCP. Finally, we found that combined loss of hepatic *Bcl6* and *Fxr* causes severe BA accumulation and toxicity due to a virtually complete loss of hepatic *Shp*, indicating that *Fxr* and *Bcl6* have independent but critical co-regulatory roles in maintaining BA homeostasis. Overall, these findings implicate BCL6 as a key integrator of FXR enterohepatic signaling and BA metabolism.

## Results

### Hepatic BCL6 and FXR Converge on Key BA Genes

**Figure 1:**
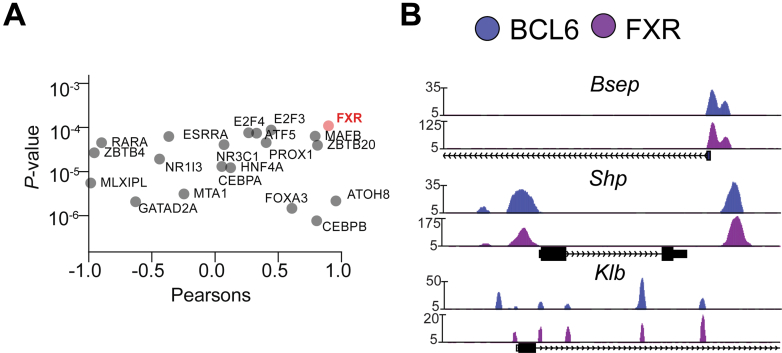
**Hepatic BCL6 is predicted to co-regulate transcription with FXR.** (*A*) Top 20 high-confidence causal transcription factors predicted by IMAGE analysis of RNA-seq and H3K27ac datasets from *Bcl6*^*fl/fl*^ and *Bcl6*^*LKO*^ livers. N = 3/group. (*B*) Representative UCSC browser tracks showing hepatic BCL6 and FXR binding at key BA regulatory genes in *Bcl6*^*fl/fl*^ liver.

To advance our understanding of BCL6 functions in the liver, we implemented a machine learning analysis pipeline, IMAGE, to integrate gene expression changes in hepatocyte *Bcl6* knockout (*Bcl6*^*LKO*^*)* mice with active vicinal enhancers and promoters.^27^ This predicted 50 high confidence BCL6 co-regulatory transcription factors, including PPARa, which we previously described to functionally converge with BCL6 in directing fatty acid metabolism.^22^ Notably, FXR emerged as a high-ranked co-regulatory factor (*P* = 10^−4^) whose activity was strongly correlated (*r* = 0.90) to gene expression changes caused by loss of *Bcl6* (Figure 1*A*). To extend these predictions, we performed FXR chromatin immunoprecipitation sequencing (ChIP-seq) in *Bcl6*^*fl/fl*^ livers and overlapped the resulting cistrome with our prior BCL6 ChIP-seq data.^22^^,^^28^ We found that BCL6 and FXR bind in close proximity along *Bsep, Shp,* and *Klb,* suggesting a potential role for BCL6 at FXR-dependent genes controlling BA metabolism (Figure 1*B*).

### Hepatic *Bcl6* Deletion Increases Circulating Sterols

**Figure 2:**
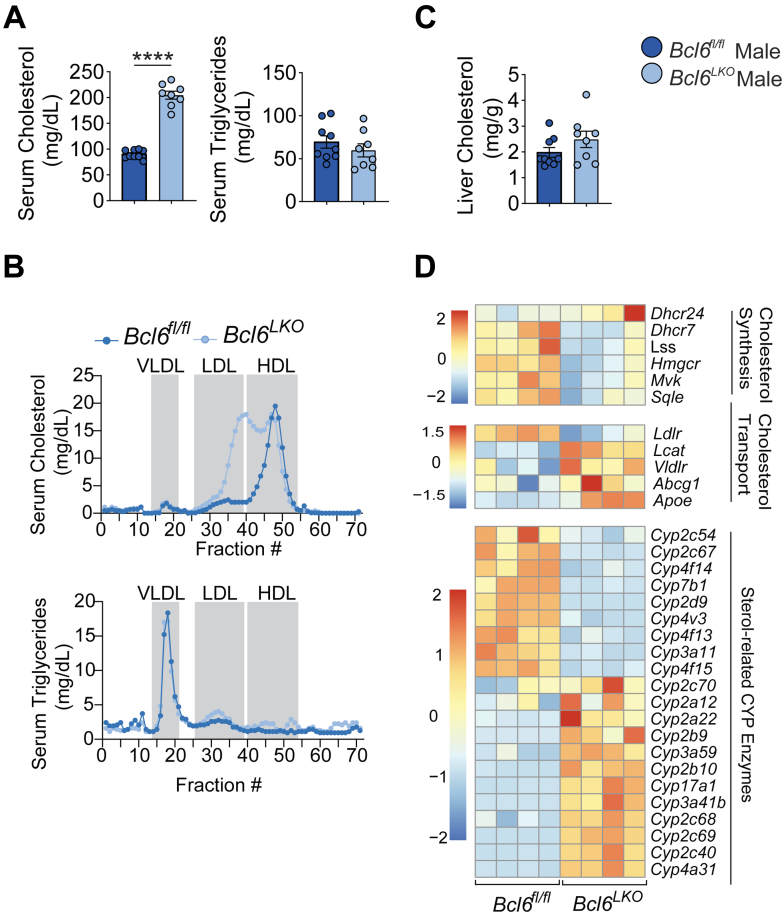
**Hepatic *Bcl6* deletion increases serum cholesterol in males.** (*A*) Serum cholesterol and triglycerides from *Bcl6*^*fl/fl*^ and *Bcl6*^*LKO*^ males. N = 8–9/group. (*B*) FPLC quantification of cholesterol and triglycerides from *Bcl6*^*fl/fl*^ and *Bcl6*^*LKO*^ pooled male serum. N = 3–4 animals/pool. (*C*) Liver cholesterol in *Bcl6*^*fl/fl*^ and *Bcl6*^*LKO*^ males. N = 8–9/group. Student’s *t*-tests were performed in (*A* and *C*) to compare means. (*D*) Heatmap of *Bcl6*^*fl/fl*^ vs *Bcl6*^*LKO*^ differentially expressed (*P*_adj_ < .05, |log_2_FC |> 0) cholesterol synthesis, transport, and sterol-related CYP genes expressed as relative RPKM from RNA-seq performed in *Bcl6*^*fl/fl*^ and *Bcl6*^*LKO*^ male livers. N = 4/group. Data are represented as mean ± SEM. ^∗^*P* < .05; ^∗∗^*P* < .01; ^∗∗∗^*P* < .001; ^∗∗∗∗^*P* < .0001.

**Figure 3:**
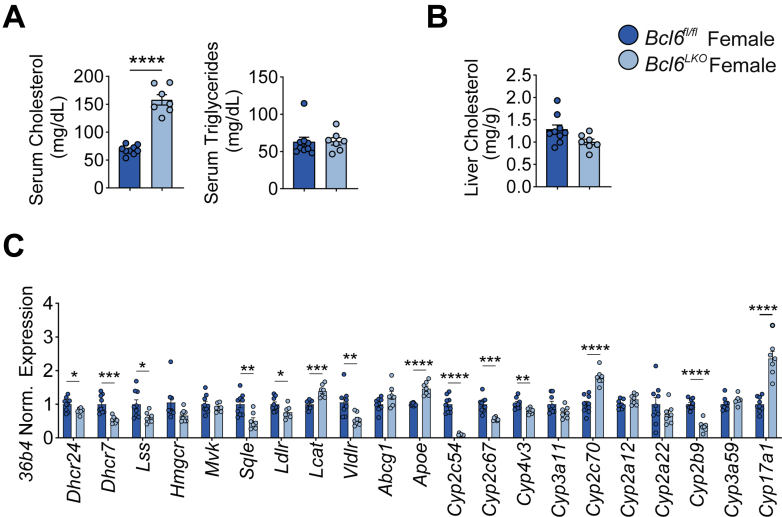
**Hepatic *Bcl6* deletion increases serum cholesterol in females.** (*A*) Serum cholesterol and triglycerides and (*B*) liver cholesterol levels in *Bcl6*^*fl/fl*^ and *Bcl6*^*LKO*^ females. N = 7–9/group. (*C*) Liver qPCR quantifying cholesterol synthesis, transport, and sterol-related CYP genes in *Bcl6*^*fl/fl*^ and *Bcl6*^*LKO*^ females. N = 7–9/group. Student’s *t*-tests or multiple Student’s *t*-tests were performed to compare means. Data are represented as mean ± SEM. ^∗^*P* < .05; ^∗∗^*P* < .01; ^∗∗∗^*P* < .001; ^∗∗∗∗^*P* < .0001.

The liver is a primary organ for sterol metabolism, synthesizing cholesterol and directing its removal through the formation of lipoproteins and BAs.^29^^,^^30^ To begin to test the impact of BCL6 on sterol metabolism, we quantified serum cholesterol and triglycerides in *Bcl6*^*LKO*^ and *Bcl6*^*fl/fl*^ male and female mice (Figure 2*A*; Figure 3*A*). Although circulating triglycerides were unchanged in both sexes, cholesterol levels were doubled in *Bcl6*^*LKO*^ animals. Evaluation of lipoproteins from pooled serum using fast protein liquid chromatography revealed an increase for both high-density lipoprotein (HDL) and LDL cholesterol fractions in these mice (Figure 2*B*), in line with findings from another report.^25^ Additionally, we found no change in *Bcl6*^*LKO*^ liver cholesterol compared with their control counterparts (Figure 2*C*; Figure 3*B*). To gain insight into transcriptional changes underlying these alterations in cholesterol, we assembled expression data for genes involved in cholesterol metabolism (Figure 2*D*) from liver transcriptomic data in our prior study of ad lib fed *Bcl6*^*LKO*^ and *Bcl6*^*fl/fl*^ male mice.^22^ We found that SREB2-dependent cholesterol biosynthetic (*Hmgcr, Mvk, Lss, Sqle, Dhcr7)* and uptake (*Ldlr)* genes were significantly reduced in mice with hepatocyte *Bcl6* ablation.^31^ In contrast, expression of genes implicated in reverse cholesterol transport was significantly increased (*Lcat*, *Abcg1*, *Apoe)* in livers of *Bcl6*^*LKO*^ mice. We also identified over 20 significantly altered CYP genes with functions in cholesterol and BA metabolism that were robustly up- or down-regulated in the livers of *Bcl6*^*LKO*^ mice.

Because hepatic *Bcl6* has sexually dimorphic expression with higher levels in males,^19^ we also examined cholesterol levels and regulation in *Bcl6*^*LKO*^ and *Bcl6*^*fl/fl*^ females. Similar to males, serum cholesterol levels were doubled in *Bcl6*^*LKO*^ females, whereas serum triglyceride levels and liver cholesterol levels were unchanged compared with female controls (Figure 3*A* and *B*). In addition, female *Bcl6*^*LKO*^ animals had reduced hepatic expression of cholesterol biosynthetic (*Dhcr24, Dhcr7, Lss, Sqle*) genes, elevated expression of cholesterol transport (*Lcat, Apoe*) genes, and changes in cholesterol-related CYP expression resembling liver alterations in *Bcl6*^*LKO*^ males (Figure 3*C*). Together, these findings showed that BCL6 regulates cholesterol metabolism and could more broadly impact liver sterol metabolism.

### Hepatic *Bcl6* Deletion Increases BA Levels and Synthesis

**Figure 4:**
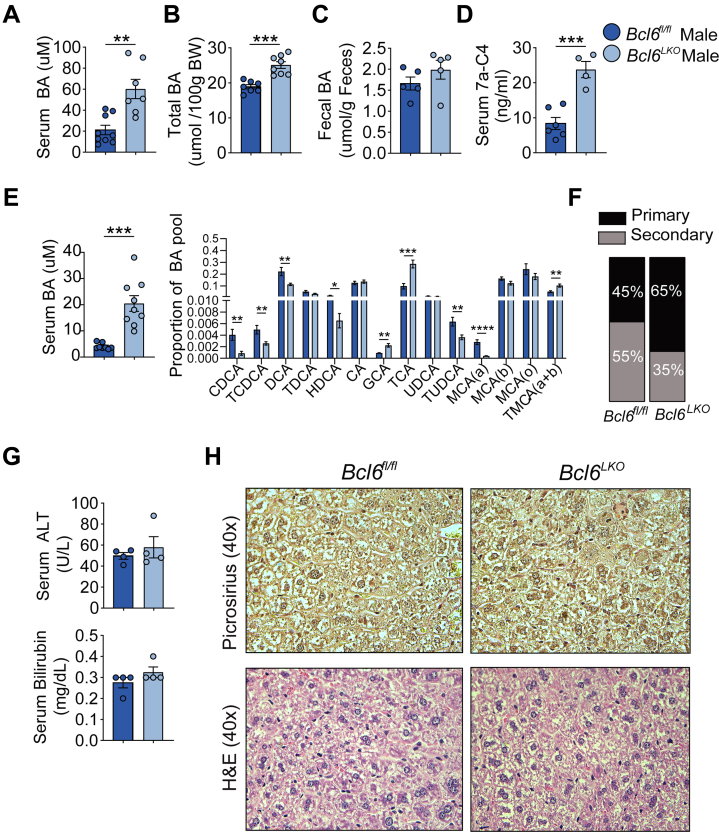
**Hepatic *Bcl6* deletion increases BA synthesis and serum/total levels in males.** (*A*) Serum BA levels in *Bcl6*^*fl/fl*^ and *Bcl6*^*LKO*^ males. N = 7–9/group. (*B*) Total BA pool quantification (including liver, gallbladder, and small intestines) in *Bcl6*^*fl/fl*^ and *Bcl6*^*LKO*^ males, expressed as umol per 100 grams of body weight. N = 7–8/group. (*C*) Total fecal BA levels in *Bcl6*^*fl/fl*^ and *Bcl6*^*LKO*^ males. N = 5/group. (*D*) Serum 7a-C4 quantification in *Bcl6*^*fl/fl*^ and *Bcl6*^*LKO*^ males. N = 4–6/group. (*E*) Total (*left*) and individual BA species in serum as proportions of total (*right*), and (*F*) percentages of primary and secondary BAs in overnight-fasted *Bcl6*^*fl/fl*^ and *Bcl6*^*LKO*^ males. N = 8–9/group. (*G*) Serum ALT and bilirubin levels in *Bcl6*^*fl/fl*^ and *Bcl6*^*LKO*^ mice. N = 4/group. (*H*) Representative Picrosirius Red and H&E staining in liver from *Bcl6*^*fl/fl*^ and *Bcl6*^*LKO*^ males. In (*A–E* and *G*), Student’s *t*-tests or multiple Student’s *t*-tests were performed to compare means. Data are represented as mean ± SEM. ^∗^*P* < .05; ^∗∗^*P* < .01; ^∗∗∗^*P* < .001; ^∗∗∗∗^*P* < .0001.

To determine whether BCL6 influences BAs, we first quantified BA levels in *Bcl6*^*fl/fl*^ and *Bcl6*^*LKO*^ male mice. We found that loss of hepatic *Bcl6* caused a ∼3× increase in serum BAs (Figure 4*A*). We also quantified the total BA pool composed of liver, gallbladder, and small intestines in these animals and found that *Bcl6*^*LKO*^ males had a ∼25% increase in total BAs compared with controls (Figure 4*B*). Fecal BA levels also trended higher but were not significantly changed in *Bcl6*^*LKO*^ males (Figure 4*C*). To test whether BCL6 controls BA synthesis, we measured serum 7a-C4, an intermediate of classical BA synthesis and marker of CYP7A1 activity.^32^ Levels of 7a-C4 were nearly 3-fold higher in *Bcl6*^*LKO*^ males compared with controls (Figure 4*D*), indicating that loss of hepatic *Bcl6* increased BA synthesis. Overnight fasting reduced circulating BA levels overall (Figure 4*E*), but *Bcl6*^*LKO*^ males continued to exhibit significantly more elevated BA levels than controls. We also examined whether BCL6 regulates the composition of BAs, finding higher proportions of classical pathway primary BAs (glycocholic acid [GCA]/taurocholic acid [TCA]) and reduced alternative pathway BAs (chenodeoxycholic acid [CDCA]/taurochenodeoxycholic acid [TCDCA]) in the serum of *Bcl6*^*LKO*^ males (Figure 4*E*; Table 1). Furthermore, loss of hepatic *Bcl6* increased the ratio of primary to secondary serum BAs in males (Figure 4*F*). We found no changes in serum alanine aminotransferase (ALT) and bilirubin or liver picrosirius red staining in *Bcl6*^*LKO*^ animals, however, indicating that their increased BA levels do not cause hepatic inflammation or fibrosis (Figure 4*G* and *H*).

**Table 1:** Serum Individual BA Concentrations (uM)

ID	Genotype	Sex	CDCA	TCDCA	DCA	TDCA	HDCA	CA	GCA	TCA	UDCA	TUDCA	MCA(a)	MCA(b)	MCA(o)	TMCA_(a+b)_	Total
1	*Bcl6*^*fl/fl*^	F	0.07	.05	0.70	0.21	0.09	0.94	0.00	0.61	0.19	0.09	0.08	2.29	0.73	0.67	6.73
2	*Bcl6*^*fl/fl*^	F	0.12	0.04	1.27	0.14	0.14	0.52	0.00	0.38	0.31	0.09	.01	1.36	1.14	0.27	5.77
3	*Bcl6*^*fl/fl*^	F	.05	0.11	2.85	1.15	0.12	3.58	0.03	2.94	0.34	0.10	0.09	2.54	2.34	1.38	17.62
4	*Bcl6*^*fl/fl*^	F	0.06	.05	1.40	0.30	0.08	1.32	.01	0.79	0.38	0.08	0.04	2.29	1.29	0.72	8.80
5	*Bcl6*^*fl/fl*^	F	0.07	0.08	2.20	0.76	0.14	2.40	.01	1.42	0.45	0.09	.05	2.48	3.08	0.93	14.16
6	*Bcl6*^*fl/fl*^	F	0.11	0.12	1.71	0.37	0.19	3.47	0.02	2.22	0.49	0.11	0.09	5.45	2.54	1.99	18.89
7	*Bcl6*^*fl/fl*^	F	0.03	0.13	0.93	0.61	0.06	1.44	.01	2.17	0.21	0.15	0.04	1.45	0.79	1.36	9.38
8	*Bcl6*^*fl/fl*^	F	0.07	0.07	2.51	0.63	0.22	2.34	.01	2.25	0.47	0.11	.05	7.84	1.60	3.77	21.93
9	*Bcl6*^*LKO*^	F	0.04	0.07	3.41	0.89	0.35	9.00	0.04	6.34	0.25	0.06	.01	4.26	6.82	2.32	33.85
10	*Bcl6*^*LKO*^	F	0.06	0.06	3.13	0.72	0.29	4.87	0.03	3.72	0.42	0.07	.01	9.04	3.74	3.48	29.63
11	*Bcl6*^*LKO*^	F	0.13	.05	1.98	0.25	0.31	6.45	0.03	3.33	0.67	0.09	.01	5.88	5.99	1.72	26.89
12	*Bcl6*^*LKO*^	F	0.07	0.11	7.83	2.17	0.31	19.10	0.09	15.20	0.41	0.09	.01	6.47	9.03	4.68	65.56
13	*Bcl6*^*LKO*^	F	0.06	0.08	3.21	0.89	0.19	3.46	0.04	5.03	0.56	0.14	.01	4.63	3.03	2.52	23.84
14	*Bcl6*^*LKO*^	F	.01	0.09	2.41	1.04	0.04	0.92	0.09	13.70	0.15	0.15	.01	1.46	1.21	5.28	26.56
15	*Bcl6*^*LKO*^	F	.01	0.07	4.23	1.45	0.04	2.92	0.06	9.37	0.28	0.13	.01	2.34	2.58	2.30	25.78
16	*Bcl6*^*LKO*^	F	0.14	0.06	5.53	0.65	0.38	3.98	0.02	4.69	0.89	0.11	.01	6.60	4.92	2.51	30.48
17	*Bcl6*^*fl/fl*^	M	0.03	.01	0.59	0.09	0.08	0.42	0.00	0.19	0.08	.01	.01	0.46	1.50	0.07	3.55
18	*Bcl6*^*fl/fl*^	M	.01	.05	0.75	0.35	0.03	1.01	.01	1.39	0.07	0.03	0.02	0.97	0.55	0.53	5.76
19	*Bcl6*^*fl/fl*^	M	0.02	0.03	2.48	0.62	0.03	0.47	.01	0.62	0.11	0.04	.01	0.69	0.76	0.28	6.15
20	*Bcl6*^*fl/fl*^	M	0.02	.01	0.65	0.09	0.09	0.30	0.00	0.15	0.04	0.02	.01	0.66	1.33	0.12	3.48
21	*Bcl6*^*fl/fl*^	M	.01	.01	0.74	0.10	0.10	0.38	0.00	0.20	.05	0.02	.01	0.76	1.27	0.17	3.81
22	*Bcl6*^*fl/fl*^	M	.01	0.02	0.58	0.15	0.10	0.41	0.00	0.25	0.04	0.03	0.02	0.75	1.01	0.23	3.60
23	*Bcl6*^*fl/fl*^	M	.01	0.02	0.65	0.22	0.03	0.52	0.00	0.50	0.04	0.03	.01	0.65	0.70	0.26	3.64
24	*Bcl6*^*fl/fl*^	M	0.02	0.02	1.06	0.21	0.04	0.57	0.00	0.24	0.06	0.03	.01	0.34	0.32	0.12	3.03
25	*Bcl6*^*LKO*^	M	.01	0.06	1.39	0.60	0.08	1.05	.05	7.17	0.09	0.06	.01	0.97	1.26	2.98	15.77
26	*Bcl6*^*LKO*^	M	.01	0.06	2.34	0.57	0.16	4.49	.05	6.82	0.34	0.08	.01	4.77	5.08	2.81	27.59
27	*Bcl6*^*LKO*^	M	0.02	0.04	1.36	0.42	0.10	0.80	0.02	2.93	0.12	.05	.01	0.81	2.35	0.83	9.85
28	*Bcl6*^*LKO*^	M	0.03	0.03	2.56	0.36	0.20	5.29	.05	3.54	0.26	0.04	.01	4.51	8.07	1.56	26.50
29	*Bcl6*^*LKO*^	M	.01	.05	1.99	0.54	0.02	1.33	0.02	4.14	0.17	0.06	.01	1.22	1.46	1.10	12.11
30	*Bcl6*^*LKO*^	M	.01	0.03	1.62	0.21	0.17	2.18	.01	2.31	0.19	0.04	.01	2.83	3.84	1.11	14.55
31	*Bcl6*^*LKO*^	M	.01	0.04	1.37	0.58	.05	2.64	0.06	6.60	0.12	0.06	.01	1.98	2.33	2.23	18.07
32	*Bcl6*^*LKO*^	M	.01	0.07	4.48	1.92	0.15	5.82	0.14	11.20	0.44	0.15	.01	4.79	4.11	4.79	38.07
33	*Bcl6*^*LKO*^	M	0.07	.05	3.05	0.76	0.24	3.37	0.04	5.99	0.41	0.10	.01	1.85	4.05	1.69	21.68

BA, bile acid; Bcl6, B-cell lymphoma 6; CA, cholic acid; CDCA, chenodeoxycholic acid; DCA, deoxycholic acid; F, female; GCA, glycocholic acid; HDCA, hyodeoxycholic acid; M, male; MCA, muricholic acid; TCA, taurocholic acid; TCDCA, taurochenodeoxycholic acid; TDCA, taurodeoxycholic acid; TMCA, tauro-α-muricholic acid; TUDCA, tauroursodeoxycholic acid; UDCA, ursodeoxycholic acid.

**Figure 5:**
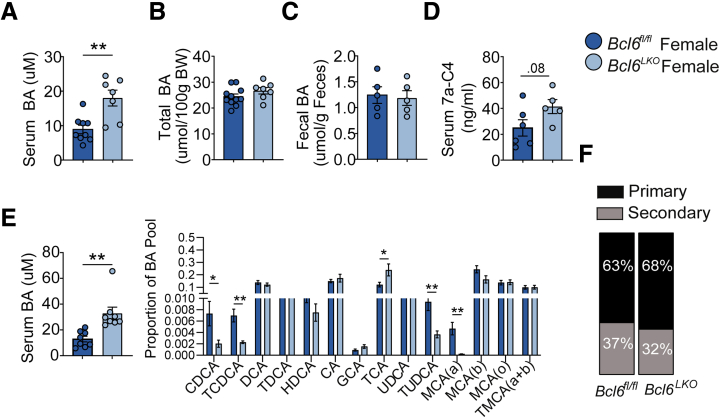
**Hepatic *Bcl6* deletion increases BA synthesis and serum levels in females.** (*A*) Serum BA levels in *Bcl6*^*fl/fl*^ and *Bcl6*^*LKO*^ females. N = 7–9/group. (*B*) Total BA pool quantification (including liver, gallbladder, and small intestines) in *Bcl6*^*fl/fl*^ and *Bcl6*^*LKO*^ females, expressed as umol per 100 grams of body weight. N = 7–11/group. (*C*) Total fecal BA levels in *Bcl6*^*fl/fl*^ and *Bcl6*^*LKO*^ females. N = 5/group. (*D*) Serum 7a-C4 quantification in *Bcl6*^*fl/fl*^ and *Bcl6*^*LKO*^ females. N = 5–6/group. (*E*) Total (*left*) and individual BA species in serum as proportions of total (*right*), and (*F*) percentages of primary and secondary BAs in overnight-fasted *Bcl6*^*fl/fl*^ and *Bcl6*^*LKO*^ females. N = 8/group. In (*A–E*), Student’s *t*-tests or multiple Student’s *t*-tests were performed to compare means. Data are represented as mean ± SEM. ^∗^*P* < .05; ^∗∗^*P* < .01; ^∗∗∗^*P* < .001; ^∗∗∗∗^*P* < .0001.

In female *Bcl6*^*LKO*^ mice, serum BAs were ∼2× increased (Figure 5*A*), but their total BA pool was unchanged compared with control females (Figure 5*B*). *Bcl6*^*LKO*^ females exhibited unchanged fecal BA levels and a trend towards increased serum 7a-C4 (*P* = .08) compared with controls (Figure 5*C* and *D*). In line with our findings in males, serum BA profiling revealed increased proportions of classical pathway primary BAs (TCA) and reduced alternative pathway BAs (CDCA/TCDCA) in *Bcl6*^*LKO*^ females (Figure 5*E*; Table 1). In contrast to males, however, the ratio of primary to secondary serum BAs was mostly unchanged in females, suggesting that hepatic *Bcl6* may be responsible for the sexual dimorphism observed in the proportion of primary vs secondary BAs (Figure 5*F*). Overall, these results indicated that hepatic *Bcl6* ablation increased the synthesis and pool of BAs in males, and it increased circulating BA levels in both sexes.

### Loss of Hepatic *Bcl6* Increases Ileal BA Signaling in Males

**Figure 6:**
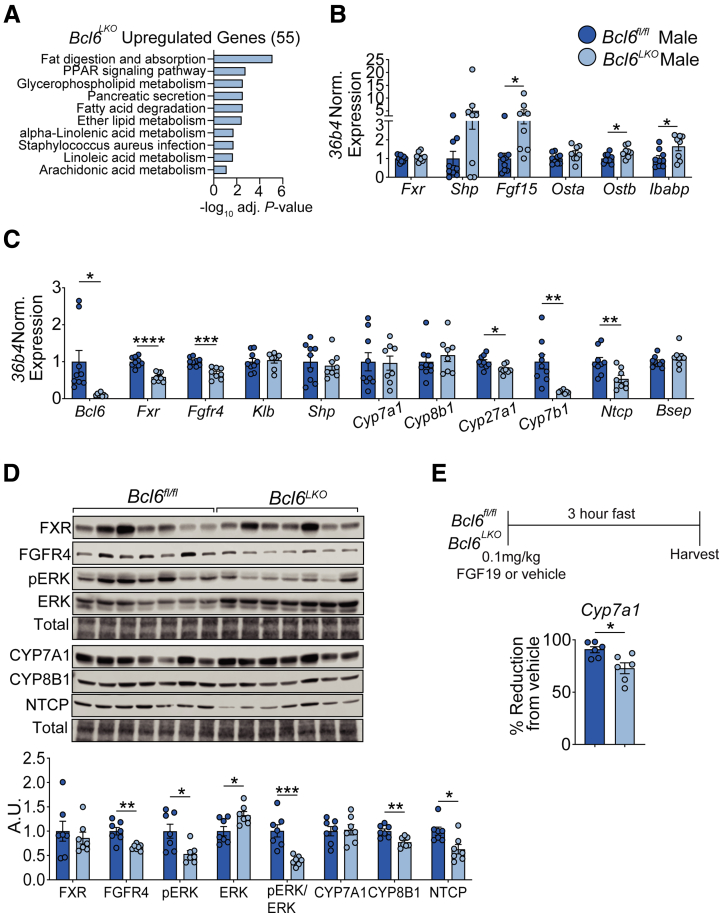
**Hepatic *Bcl6* deletion activates ileal BA signaling while reducing liver sensitivity to FGF15/19 in males.** (*A*) KEGG pathway analysis of ileal *Bcl6*^*LKO*^ upregulated genes (log_2_FC > 0, *P*_adj_ < .05) from RNA-seq performed in *Bcl6*^*fl/fl*^ and *Bcl6*^*LKO*^ male ileum. N = 4/group. (*B*) qPCR of FXR target genes in ileum and (*C*) liver from *Bcl6*^*fl/fl*^ and *Bcl6*^*LKO*^ males. N = 8–9/group. (*D*) Western blot of BA feedback signaling markers from *Bcl6*^*fl/fl*^ and *Bcl6*^*LKO*^ male liver. N = 7/group. (*E*) qPCR of hepatic *Cyp7a1* after treatment with FGF19, expressed as % reduction from respective *Bcl6*^*fl/fl*^ or *Bcl6*^*LKO*^ vehicle-treated expression. N = 6/group. In (*B–D*), multiple Student’s *t*-tests were performed to compare means. Data are represented as mean ± SEM. ^∗^*P* < .05; ^∗∗^*P* < .01; ^∗∗∗^*P* < .001; ^∗∗∗∗^*P* < .0001.

To elucidate the mechanism by which loss of BCL6 increases BA synthesis, we next examined the impact of BCL6 on BA feedback signaling. FXR controls BA homeostasis through its combined actions in the liver and intestine. In the ileum, BA activation of FXR induces FGF15 (FGF19 in humans) expression and release. FGF15 then binds the FGFR4/beta-Klotho (KLB) receptor complex on hepatocytes, which suppresses transcription of the rate-limiting BA synthetic enzyme *Cyp7a1*.^12^ To determine the role of BCL6 in this feedback pathway, we queried transcriptional changes in *Bcl6*^*fl/fl*^ and *Bcl6*^*LKO*^ ileum using RNA sequencing (RNA-seq). Notably, we found that genes differentially expressed in the ilea of *Bcl6*^*LKO*^ male mice were highly enriched in pathways involved in ‘Fat digestion and absorption’ (Figure 6*A*), suggesting a possible increase in BA signaling consistent with their elevated BA pools (Figure 4*B*). To confirm this, we interrogated the expression of key FXR target genes in the ilea of *Bcl6*^*LKO*^ males compared with controls. We detected 5-fold elevated *Fgf15,* ∼1.5-fold elevated *Ostb* and *Ibabp*, and trending increases in *Shp* levels in ilea of *Bcl6*^*LKO*^ mice (Figure 6*B*). These findings indicated that hepatic *Bcl6* ablation enhances ileal FXR signaling and FGF15/19 levels in males.

### Hepatic *Bcl6* Deletion Reduces Sensitivity to FGF15 in Males

Elevated FGF15/19 levels should efficiently shut down BA synthesis by repressing *Cyp7a1*. Counterintuitively, we found raised ileal *Fgf15* (Figure 6*B*) along with increased bile acid synthesis and serum and total BA levels in *Bcl6*^*LKO*^ males (Figure 4*A, B* and *D*). Based upon these incongruent findings, we hypothesized that BCL6 promotes FGF15/19 signaling. FGF15/19 signals in the liver by binding to FGFR4/KLB receptor, triggering pERK activation and increased *Shp* expression, causing repression of *Cyp7a1* transcription. To examine the role for BCL6 in this pathway, we first quantified levels of the hepatic FGF15 receptor FGFR4/KLB. Although we observed no change in *Klb* expression with loss of *Bcl6*, we found reduced FGFR4 mRNA and protein levels in *Bcl6*^*LKO*^ male livers (Figure 6*C* and *D*). Consistent with reduced FGFR4 signaling, we also observed decreased pERK levels, and no change in *Shp* or *Cyp7a1* expression in *Bcl6*^*LKO*^ males compared with controls despite their high ileal *Fgf15* (Figure 6*C* and *D*). To definitively determine whether BCL6 modulates hepatic sensitivity to FGF15/19, we injected *Bcl6*^*fl/fl*^ and *Bcl6*^*LKO*^ males with recombinant FGF19 and then measured the resulting reduction in *Cyp7a1* expression after 3 hours compared with vehicle controls. Food was removed upon FGF19 administration to avoid confounding effects from endogenous meal-induced FGF15 during treatment.^33^^,^^34^ Although FGF19 suppressed *Cyp7a1* expression by 90% in control livers, FGF19 treatment reduced *Cyp7a1* levels by only 73% in *Bcl6*^*LKO*^ mice (Figure 6*E*). Together, these findings reflected that *Bcl6*^*LKO*^ livers are moderately resistant to FGF15/19-mediated signaling.

### Females Lacking Hepatic *Bcl6* Have Unchanged Ileal BA Signaling and Reduced FGF15 Sensitivity

**Figure 7:**
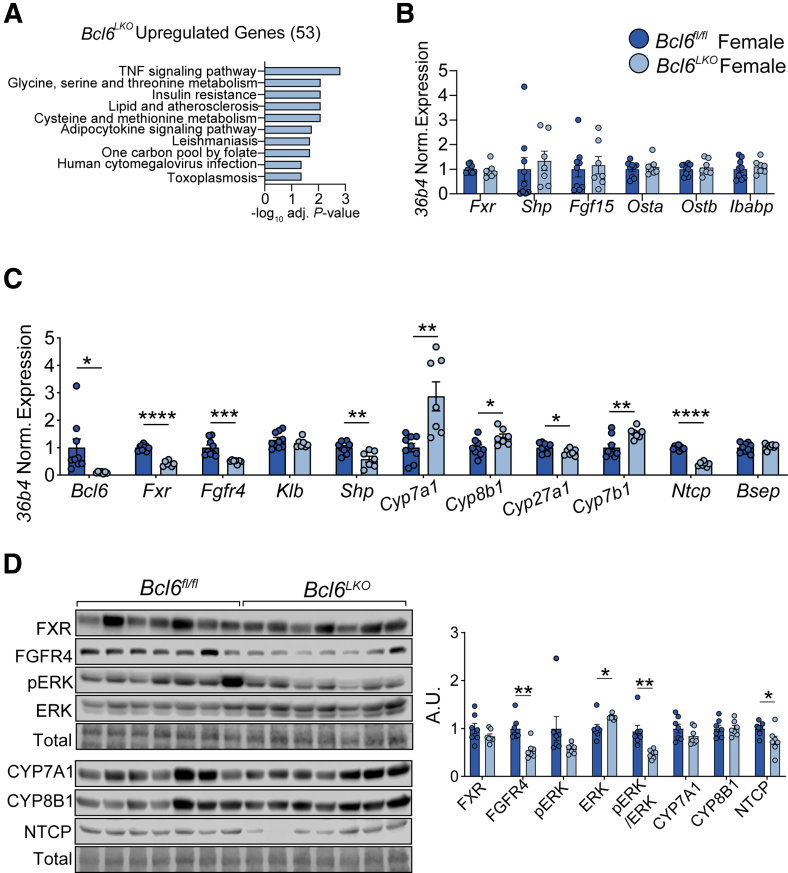
**Hepatic *Bcl6* deletion activates hepatic *Cyp7a1* in females.** (*A*) KEGG pathway analysis of ileal *Bcl6*^*LKO*^ upregulated genes (log_2_FC > 0, *P*_adj_ < .05) from RNA-seq performed in *Bcl6*^*fl/fl*^ and *Bcl6*^*LKO*^ female ileum. N = 4/group. (*B*) qPCR of FXR target genes in ileum and (*C*) liver from *Bcl6*^*fl/fl*^ and *Bcl6*^*LKO*^ females. N = 7–9/group. (*D*) Western blot of BA feedback signaling markers from *Bcl6*^*fl/fl*^ and *Bcl6*^*LKO*^ female liver. N = 7/group. In (*B–D*), multiple Student’s *t*-tests were performed to compare means. Data are represented as mean ± SEM. ^∗^*P* < .05; ^∗∗^*P* < .01; ^∗∗∗^*P* < .001; ^∗∗∗∗^*P* < .0001.

Interestingly, when we examined gene expression in the ilea of female mice, we did not find enrichment of lipid absorption pathways or increased expression of FXR target genes, including *Fgf15,* with loss of hepatic *Bcl6*. (Figure 7*A* and *B*). These findings, along with the unchanged BA pools in *Bcl6*^*LKO*^ females, suggest that *Bcl6*^*LKO*^ females lack the increased ileal BA signaling found in male *Bcl6*^*LKO*^ mice. However, *Bcl6*^*LKO*^ females exhibited reduced hepatic FGFR4 transcript and protein expression, similar to findings in *Bcl6*^*LKO*^ males and suggesting they may also have reduced sensitivity to FGF15-mediated *Cyp7a1* repression (Figure 7*C* and *D*). Consistent with their reduced FGFR4 levels, female *Bcl6*^*LKO*^ livers had reduced pERK signaling, reduced *Shp* expression, and increased *Cyp7a1* and *Cyp8b1* (Figure 7*C* and *D*). Overall, these findings suggest a model in which both male and female *Bcl6*^*LKO*^ mice are resistant to FGF15-mediated *Cyp7a1* repression due to reduced FGFR4 expression. In *Bcl6*^*LKO*^ males, the elevated *Fgf15* is sufficient to maintain WT *Cyp7a1* levels, while *Bcl6*^*LKO*^ females, which do not have increased ileal *Fgf15* expression, have elevated *Cyp7a1* levels.

### Hepatic BCL6 Deletion Reduces NTCP Expression in Both Sexes

We also tested whether BCL6 controls expression of BA transporters, which are known to influence circulating BA levels.^35^^,^^36^ The BA efflux pump *Bsep* is responsible for the majority of BA export from the liver, whereas the bulk of BA re-uptake into the liver from circulation is mediated by the transporter NTCP *(Slc10a1)*.^13^^,^^37^ We observed no change in *Bsep* expression in males and females with loss of *Bcl6*, but we did detect a ∼40% to 50% reduction in NTCP mRNA and protein levels in both sexes, suggesting that the increased serum BA levels observed in *Bcl6*^*LKO*^ animals may be partially due to reduced BA re-uptake into the liver (Figure 6*C* and *D*; Figure 7*C* and *D*).

### Hepatic BCL6 Deletion Reduces Alternative BA Synthesis Enzyme Expression

Based on our observation that loss of hepatic *Bcl6* increased serum BAs and the proportion of classical BAs (GCA/TCA) relative to alternative pathway BAs (CDCA/TCDCA) (Figure 4*E*; Figure 5*E*), we tested the expression of CYP enzymes that determine levels of individual BA species. CYP8B1 catalyzes the conversion to cholic acid (CA) in the classical BA synthesis pathway and is critical for controlling relative levels of CA vs CDCA.^38^ With loss of *Bcl6* in males, we observed no changes in *Cyp8b1* expression despite a 22% decrease of its protein level (Figure 6*C* and *D*). The enzymes CYP27A1 and CYP7B1 catalyze reactions in the alternative BA synthesis pathway, which generates CDCA.^39^ Consistent with an elevated ratio of CA compared with CDCA in the serum of *Bcl6*^*LKO*^ males, we found 20% reduced *Cyp27a1* and 80% reduced *Cyp7b1* in the livers of *Bcl6*^*LKO*^ males compared with controls (Figure 6*C* and *D*). *Bcl6*^*LKO*^ females showed a slight increase in *Cyp8b1* gene expression, but unchanged protein levels. Similar to males, female *Bcl6*^*LKO*^ livers had reduced *Cyp27a1* but elevated levels of *Cyp7b1*, a known sexually dimorphic CYP enzyme (Figure 7*C* and *D*).^21^ These data suggest that BCL6 maintains the balance between classical and alternative BA synthesis by upregulating alternative pathway enzymes.

### Dual Loss of FXR and Hepatic BCL6 (*Bcl6*^*LKO*^*Fxr*^*KO*^) Results in Cholestatic Liver Disease

**Figure 8:**
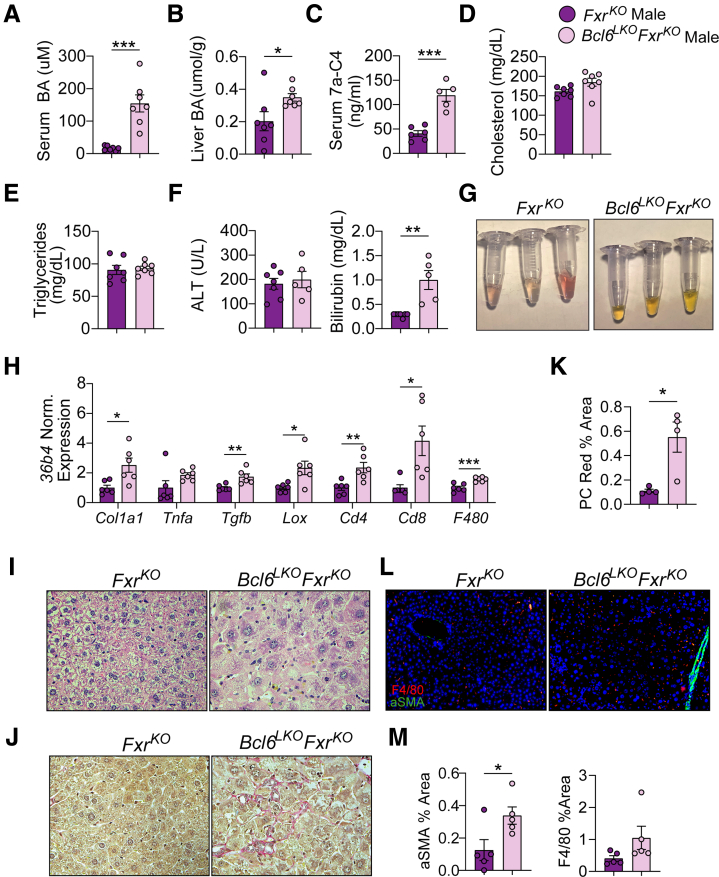
**Dual deletion of *Fxr* and hepatic *Bcl6* causes BA overload and liver damage in males.** (*A*) Serum BAs, (*B*) liver BAs, (*C*) serum 7a-C4, (*D*) serum cholesterol, (*E*) serum triglycerides, and (*F*) serum ALT and bilirubin in *Fxr*^*KO*^ and *Bcl6*^*LKO*^*Fxr*^*KO*^ males. N = 5–7/group. (*G*) Representative image of serum from *Fxr*^*KO*^ and *Bcl6*^*LKO*^*Fxr*^*KO*^ males. (*H*) Liver qPCR of *Col1a1* and immune markers in *Fxr*^*KO*^ and *Bcl6*^*LKO*^*Fxr*^*KO*^ males. N = 6/group. (*I*) Representative H&E stained (40×) and (*J*) Picrosirius Red stained (40×) images from *Fxr*^*KO*^ and *Bcl6*^*LKO*^*Fxr*^*KO*^ male livers. (*K*) Picrosirius Red quantification expressed as % total area in *Fxr*^*KO*^ and *Bcl6*^*LKO*^*Fxr*^*KO*^ male livers. Each data point is the average of 3 images/liver. N = 4/group. (*L*) Representative (20×) images of aSMA (*green*) and F4/80 (*red*) immunofluorescence in *Fxr*^*KO*^ and *Bcl6*^*LKO*^*Fxr*^*KO*^ male livers. (*M*) aSMA and F4/80 image quantification expressed as % total area in *Fxr*^*KO*^ and *Bcl6*^*LKO*^*Fxr*^*KO*^ male liver. Each data point is the average of 4 images/liver. N = 5/group. In (*A–F, H, K* and *M*) Student’s *t*-tests or multiple Student’s *t*-tests were performed to compare means. Data are represented as mean ± SEM. ^∗^*P* < .05; ^∗∗^*P* < .01; ^∗∗∗^*P* < .001; ^∗∗∗∗^*P* < .0001.

The interrelationship between BCL6 and FXR gene regulation in the enterohepatic system prompted us to test their combined impact on BA homeostasis. To this end, we generated mice lacking both hepatic *Bcl6* and whole body *Fxr* (*Bcl6*^*LKO*^*Fxr*^*KO*^) and compared these with animals with loss of *Fxr* alone (*Fxr*^*KO*^). Although singular loss of *Bcl6* resulted in a ∼3-fold elevation in serum BA levels compared with control males (Figure 4*A*), *Bcl6*^*LKO*^*Fxr*^*KO*^ males had levels that were nearly 10-fold elevated compared with *Fxr*^*KO*^ animals (Figure 8*A*). *Bcl6*^*LKO*^*Fxr*^*KO*^ males also had elevated liver BA levels, suggesting possible cholestasis (Figure 8*B*). Correspondingly, serum 7a-C4 levels were nearly doubled in males with combined loss of *Fxr* and hepatocyte *Bcl6* compared with mice with deficiency of *Fxr* alone, indicating that FXR and hepatocyte BCL6 additively suppress BA synthesis (Figure 8*C*). Interestingly, *Bcl6*^*LKO*^*Fxr*^*KO*^ males had similar serum cholesterol and triglyceride levels compared with their *Fxr*^*KO*^ counterparts (Figure 8*D* and *E*). This indicated that FXR may play a role in the elevated cholesterol levels observed with loss of hepatic *Bcl6* (Figure 2*A*), and previous work revealed that *Fxr*-deficient mice have elevated serum cholesterol levels, further suggesting that BCL6 and FXR co-regulate sterol levels.^40^

In line with the large elevations in liver BAs and serum 7a-C4 levels in *Bcl6*^*LKO*^*Fxr*^*KO*^ mice, we detected evidence of cholestatic liver damage in these mice, including increased serum bilirubin, visibly apparent yellowing of serum, and 2- to 4-fold elevated hepatic expression of pro-fibrotic (*Col1a1, Tgfb, Lox),* proinflammatory (*Tnfa*), and immune cell marker (*Cd4, Cd8, F4/80*) genes (Figure 8*F–H*). Furthermore, we observed hepatocellular hypertrophy, 5-fold increased liver picrosirius red collagen staining, 2-fold increased alpha smooth muscle actin, and a trend towards increased F4/80 macrophage staining in *Bcl6*^*LKO*^*Fxr*^*KO*^ livers compared with their *Fxr*^*KO*^ counterparts (Figure 8*I–M*).

**Figure 9:**
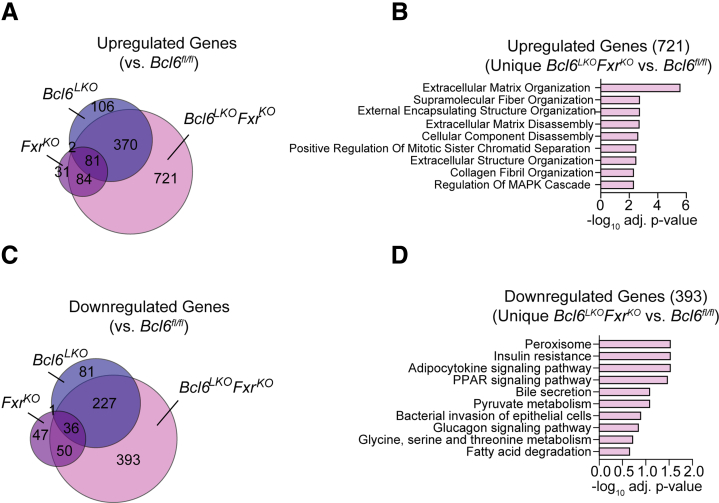
**Combined loss of *Fxr* and hepatic *Bcl6* upregulates pro-fibrotic genes.** (*A*) Venn diagram showing common and unique upregulated genes (*P*_adj_ < .05; log_2_FC >0.5) in male *Fxr*^*KO*^ , *Bcl6*^*LKO*^, or *Bcl6*^*LKO*^*Fxr*^*KO*^ livers compared with *Bcl6*^*fl/fl*^ livers. (*B*) Top GO pathways of genes uniquely upregulated in *Bcl6*^*LKO*^*Fxr*^*KO*^ vs *Bcl6*^*fl/fl*^ livers. (*C*) Venn diagram of common and unique downregulated genes (*P*_adj_ < .05; log_2_FC <0.5) in male *Fxr*^*KO*^, *Bcl6*^*LKO*^, or *Bcl6*^*LKO*^*Fxr*^*KO*^ livers compared with *Bcl6*^*fl/fl*^ livers. (*D*) Top GO pathways of genes uniquely downregulated in male *Bcl6*^*LKO*^*Fxr*^*KO*^ vs *Bcl6*^*fl/fl*^ livers. N = 4/group.

To better understand the transcriptional pathways co-regulated by BCL6 and FXR, we performed RNA-seq in *Fxr*^*KO*^ and *Bcl6*^*LKO*^*Fxr*^*KO*^ male livers and compared this with data from *Bcl6*^*fl/fl*^ and *Bcl6*^*LKO*^ males. We identified 721 genes uniquely upregulated in *Bcl6*^*LKO*^*Fxr*^*KO*^ compared with *Bcl6*^*fl/fl*^ mice, whose expression was unaffected by single deletion of either *Fxr* or hepatic *Bcl6* (Figure 9*A*). Gene Ontology enrichment analysis revealed that these uniquely upregulated genes were highly linked to pathways including ‘Extracellular Matrix Organization’ and ‘Collagen Fibril Organization,’ in line with the elevated liver *Col1a1* expression and picrosirius red staining observed in *Bcl6*^*LKO*^*Fxr*^*KO*^ mice compared to *Fxr*^*KO*^ counterparts (Figure 9*B*). Furthermore, we identified 393 uniquely downregulated liver genes in *Bcl6*^*LKO*^*Fxr*^*KO*^ compared with *Bcl6*^*fl/fl*^ and single knockout animals. These included genes implicated in ‘Bile Secretion,’ consistent with the cholestasis observed in livers of *Bcl6*^*LKO*^*Fxr*^*KO*^ mice (Figure 9*C* and *D*).

**Figure 10:**
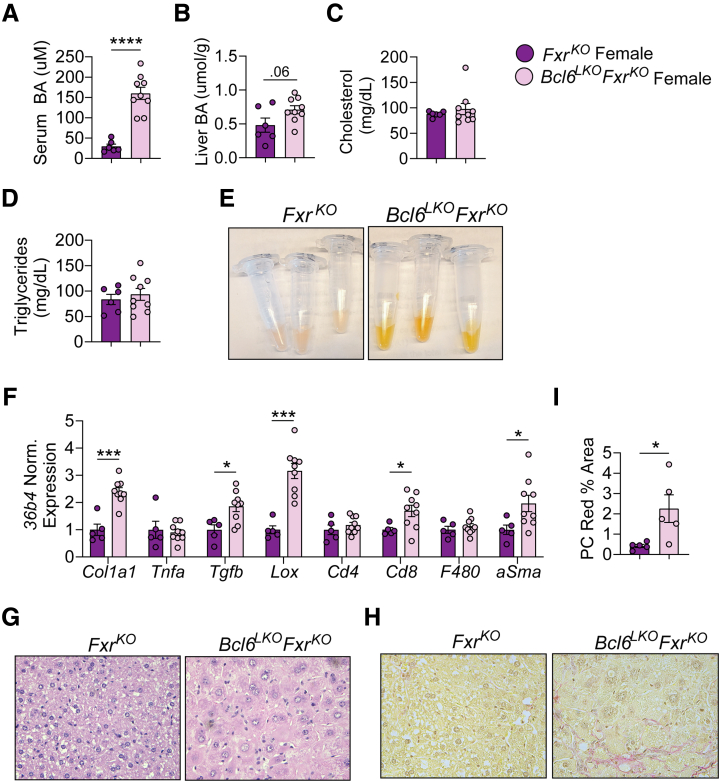
**Dual deletion of *Fxr* and hepatic *Bcl6* causes BA overload and liver damage in females.** (*A*) Serum BAs, (*B*) liver BAs, (*C*) serum cholesterol, and (*D*) serum triglycerides in *Fxr*^*KO*^ and *Bcl6*^*LKO*^*Fxr*^*KO*^ females. N = 6–9/group. (*E*) Representative image of serum from *Fxr*^*KO*^ and *Bcl6*^*LKO*^*Fxr*^*KO*^ females. (*F*) Liver qPCR of *Col1a1* and immune markers in *Fxr*^*KO*^ and *Bcl6*^*LKO*^*Fxr*^*KO*^ females. N = 5–9/group. (*G*) Representative H&E stained (40×) and (*H*) Picrosirius Red stained (40×) images from *Fxr*^*KO*^ and *Bcl6*^*LKO*^*Fxr*^*KO*^ female livers. (*I*) Picrosirius Red quantification expressed as % total area in *Fxr*^*KO*^ and *Bcl6*^*LKO*^*Fxr*^*KO*^ female liver. Each data point is the average of 3 images/liver. N = 5/group. In (*A–D, F,* and *I*), Student’s *t*-tests or multiple Student’s *t*-tests were performed to compare means. Data are represented as mean ± SEM. ^∗^*P* < .05; ^∗∗^*P* < .01; ^∗∗∗^*P* < .001; ^∗∗∗∗^*P* < .0001.

In females, dual-loss of hepatic *Bcl6* and *Fxr* similarly caused ∼5-fold elevations in serum BAs, a strong trend towards increased liver BAs (*P* = .06), and no change in serum cholesterol or triglycerides compared with *Fxr*^*KO*^ females (Figure 10*A–D*). Like *Bcl6*^*LKO*^*Fxr*^*KO*^ males, *Bcl6*^*LKO*^*Fxr*^*KO*^ females exhibited jaundiced serum, increased expression of pro-fibrotic (*Col1a1, aSma*) and inflammatory/immune marker genes (*Tgfb, Lox, Cd8*), hepatocyte ballooning, and increased liver picrosirius red staining (Figure 10*E–I*). Overall, these data indicated that BCL6 and FXR co-regulate BA production and, in the absence of *Fxr*, BCL6 is critical to restrain synthesis and hepatotoxic overload of BAs.

### Dual Deletion of *Fxr* and Hepatic *Bcl6* Causes Profound Loss of SHP-mediated Repression and BA Overload

**Figure 11:**
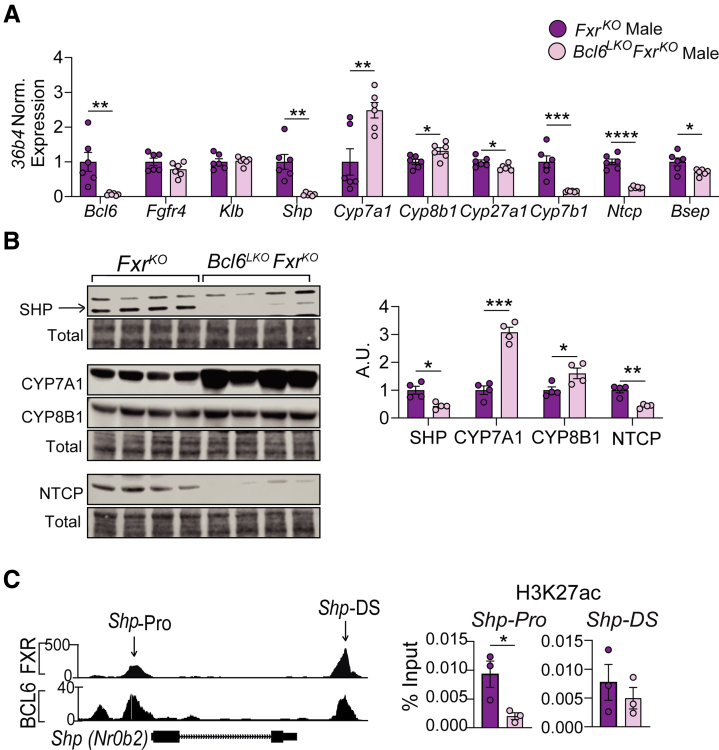
**Dual deletion of *Fxr* and hepatic *Bcl6* in males results in loss of SHP.** (*A*) qPCR and (*B*) Western blots of BA regulatory targets in *Fxr*^*KO*^ and *Bcl6*^*LKO*^*Fxr*^*KO*^ male livers. N = 4–6/group. (*C*) (*Left*) UCSC browser tracks at the *Shp (Nr0b2)* locus, showing FXR (*top*) and BCL6 (*bottom*) binding sites in control male livers. *Arrows* annotate the Shp promoter (*Shp-Pro*) and Shp downstream enhancer (*Shp-DS*) regions. (*Righ*t) H3K27ac ChIP qPCR at the *Shp-Pro* and *Shp-DS* regions in *Fxr*^*KO*^ and *Bcl6*^*LKO*^*Fxr*^*KO*^ male livers, expressed as percent of input chromatin. N = 3/group. Student’s *t*-tests or multiple Student’s *t*-tests were performed to compare means. Data are represented as mean ± SEM. ^∗^*P* < .05; ^∗∗^*P* < .01; ^∗∗∗^*P* < .001; ^∗∗∗∗^*P* < .0001.

**Figure 12:**
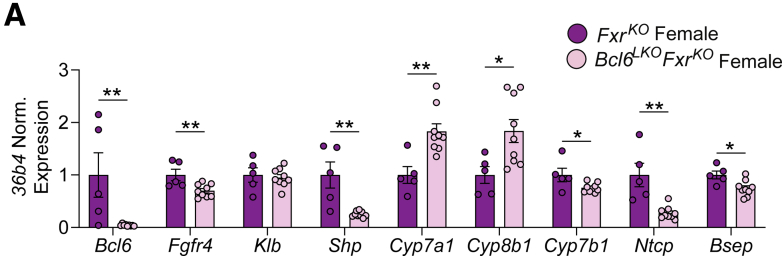
**Dual deletion of *Fxr* and hepatic *Bcl6* in females results in loss of SHP.** (*A*) qPCR of BA regulatory targets in *Fxr*^*KO*^ and *Bcl6*^*LKO*^*Fxr*^*KO*^ female livers. N = 5–9/group. Multiple Student’s *t*-tests were performed to compare means. Data are represented as mean ± SEM. ^∗^*P* < .05; ^∗∗^*P* < .01; ^∗∗∗^*P* < .001; ^∗∗∗∗^*P* < .0001.

We next examined the mechanism driving the massive elevation in BAs observed with co-deletion of *Fxr* and hepatocyte *Bcl6*. Hepatic *Shp (Nr0b2)* is activated through both liver-intrinsic and enterohepatic mechanisms directed by FXR.^12^ Remarkably, we found that *Bcl6*^*LKO*^*Fxr*^*KO*^ males and females had a profound loss of liver *Shp m*RNA and 57% reduced small heterodimer partner (SHP) protein levels (Figure 11*A* and *B*; Figure 12*A*). To better understand the large reduction in *Shp*, we interrogated chromatin activity at the *Shp* locus by performing ChIP-quantitative polymerase chain reaction (qPCR) with histone 3 lysine 27 acetyl (H3K27ac) antibody (Figure 11*C*). In *Bcl6*^*LKO*^*Fxr*^*KO*^ mice, we detected an ∼80% reduction of H3K27ac at the *Shp* promoter site where BCL6 and FXR each bind and a trending reduction in H3K27ac levels at an enhancer site just downstream of the *Shp* locus where BCL6 and FXR likewise bind, consistent with the loss of *Shp* transcription (Figure 11*C*). In addition, mRNA and protein levels of the SHP target genes CYP8B1 and CYP7A1 were upregulated ∼1- to 3-fold in *Bcl6*^*LKO*^*Fxr*^*KO*^ mice compared to animals lacking *Fxr* alone (Figure 11*A* and *B*; Figure 12*A*), reflecting a loss of SHP-mediated repression. *Bcl6*^*LKO*^*Fxr*^*KO*^ animals exhibited reduced liver *Ntcp, Cyp27a1*, and *Cyp7b1,* paralleling the reductions observed with loss of *Bcl6* alone and suggesting these genes are controlled by BCL6 independently of FXR (Figure 11*A* and *B*; Figure 12*A*). Interestingly, *Bcl6*^*LKO*^*Fxr*^*KO*^ males did not exhibit reduced *Fgfr4* expression compared with *Fxr*^*KO*^ males, whereas this reduction was preserved in females. This suggested that hepatic *Bcl6* may indirectly regulate *Fgfr4* in a manner that is sexually dimorphic in the absence of *Fxr* (Figure 11*A*; Figure 12*A*). Combined loss of hepatic *Bcl6* and *Fxr* also reduced expression of the BA exporter *Bsep*, consistent with the elevated liver BAs and cholestatic damage we detected in *Bcl6*^*LKO*^*Fxr*^*KO*^ animals (Figure 11*A*; Figure 12*A*). These findings established BCL6 as a key partner for hepatic FXR to cooperatively maintain *Shp* expression.

**Figure 13:**
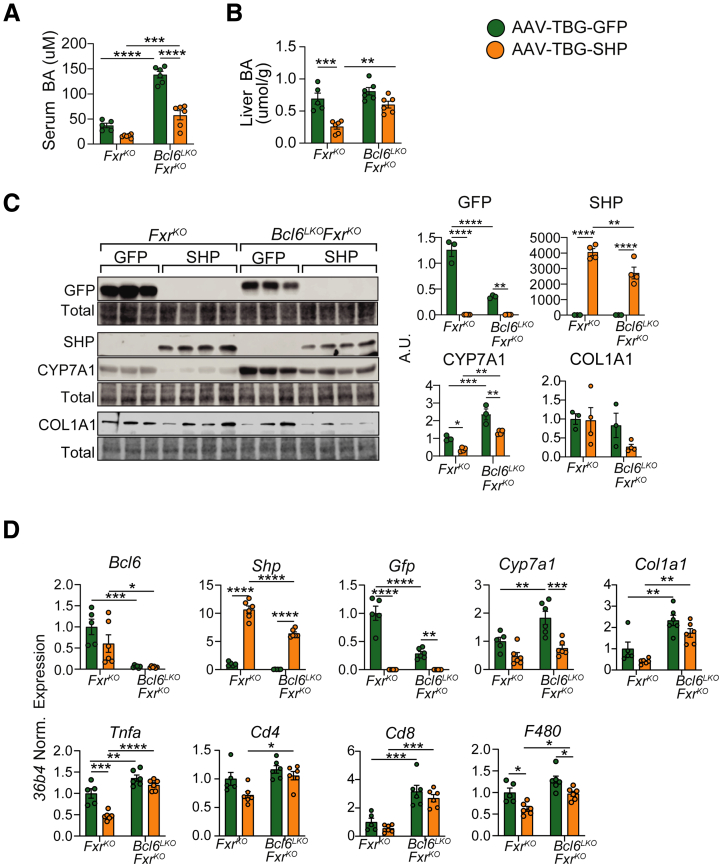
**SHP is required for BCL6-FXR co-suppression of CYP7A1 and BAs in males.** (*A*) Serum, (*B*) liver total BAs, (*C*) liver western blot of GFP, SHP, CYP7A1, and COL1A1, and (*D*) liver qPCR of *Bcl6, Shp, Gfp*, *Cyp7a1,* and immune marker genes in *Fxr*^*KO*^ and *Bcl6*^*LKO*^*Fxr*^*KO*^ males treated with AAV-TBG-GFP or AAV-TBG-SHP for 4 weeks. N = 3–6/group. Two-way ANOVA with Holm-Sidak’s post-hoc testing was performed to compare effects of AAV-treatment, genotype, and their interaction on means. Data are represented as mean ± SEM. ^∗^*P* < .05; ^∗∗^*P* < .01; ^∗∗∗^*P* < .001; ^∗∗∗∗^*P* < .0001.

To definitively determine whether the large increases in CYP7A1 and serum BAs were due to the drop in liver *Shp* expression in *Bcl6*^*LKO*^*Fxr*^*KO*^ mice, we transduced *Bcl6*^*LKO*^*Fxr*^*KO*^ and *Fxr*^*KO*^ males with adeno-associated virus serotype 8 (AAV8) expressing either *Gfp* (AAV8-TBG-GFP) or *Shp* (AAV8-TBG-SHP) from the thyroid binding globulin promoter and examined their livers 4 weeks post-AAV injection. We found that overexpression of *Shp* in the liver was sufficient to suppress serum BAs in *Bcl6*^*LKO*^*Fxr*^*KO*^ mice down to quantities observed in *Fxr*^*KO*^ mice treated with green-fluorescent protein (GFP)-negative control virus (Figure 13*A*). Similarly, *Shp* overexpression caused a trending decrease in liver BA levels in *Bcl6*^*LKO*^*Fxr*^*KO*^ mice (Figure 13*B*). In line with reduced serum BA levels, AAV-TBG-SHP treatment reduced liver CYP7A1 gene and protein expression to levels found in *Fxr*^*KO*^ mice treated with GFP (Figure 13*C* and *D*). We also observed a trend towards reduced liver COL1A1 mRNA and protein expression in *Shp*-transduced *Bcl6*^*LKO*^*Fxr*^*KO*^ mice and a slight but significant reduction in their *F4/80* expression, suggesting that SHP overexpression may attenuate hepatic damage in *Bcl6*^*LKO*^*Fxr*^*KO*^ mice (Figure 13*C* and *D*). Overall, these data indicate that SHP is required for BCL6 and FXR co-repression of *Cyp7a1* and BAs.

### Extrahepatic FXR Coordinates with Hepatocyte BCL6 to Suppress BAs

**Figure 14:**
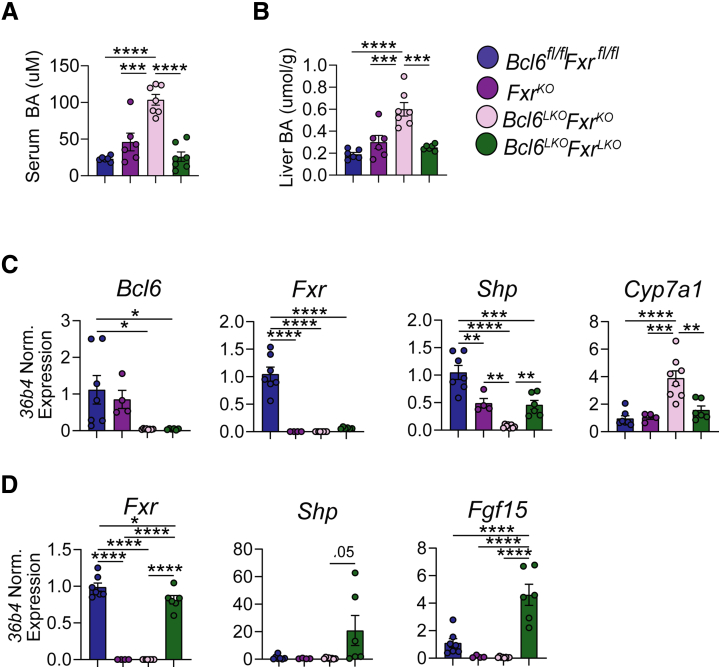
**Hepatic *Fxr* is not required for FXR-BCL6 co-regulation of BAs.** (*A*) Serum total BAs, (*B*) liver total BAs, (*C*) liver qPCR of *Bcl6, Fxr, Shp,* and *Cyp7a1*, and (*D*) ileal qPCR of *Fxr, Shp,* and *Fgf15* in *Bcl6*^*fl/fl*^*Fxr*^*fl/fl*^*, Fxr*^*KO*^*, Bcl6*^*LKO*^*Fxr*^*KO*^, and *Bcl6*^*LKO*^*Fxr*^*LKO*^ males. N = 4–8/group. One-way ANOVA with Holm-Sidak’s post-hoc testing was performed to compare means. Data are represented as mean ±SEM. ^∗^*P* < .05; ^∗∗^*P* < .01; ^∗∗∗^*P* < .001; ^∗∗∗∗^*P* < .0001.

Hepatic SHP inhibits *Cyp7a1* expression and BA synthesis through: (1) an ileal-liver negative feedback pathway involving FGF15-mediated induction of *Shp*; and (2) a liver-intrinsic pathway by which hepatic FXR activates *Shp* expression. Because both ileal and liver FXR play a part in BA feedback signaling, we sought to determine the relative contribution of hepatocyte vs global *Fxr* deletion in driving the massive BA elevation observed in *Bcl6*^*LKO*^*Fxr*^*KO*^ mice. To test this, we crossed *Bcl6*^*LKO*^ mice with *Fxr*^*fl/fl*^ mice to generate hepatocyte-specific double knockout (*Bcl6*^*LKO*^*Fxr*^*LKO*^) mice and compared these with *Fxr* full-body knockout counterparts (*Bcl6*^*LKO*^*Fxr*^*KO*^ and *Fxr*^*KO*^ mice). We found that *Bcl6*^*LKO*^*Fxr*^*LKO*^ mice, which lack *Fxr* in liver but express it in ileum, had serum and liver BA levels resembling those of wild-type control (*Bcl6*^*fl/fl*^*Fxr*^*fl/fl*^) mice (Figure 14*A* and *B*). Consistent with their reduced BA levels, we observed increased *Shp* and reduced *Cyp7a1* in the livers of double hepatocyte knockout mice (*Bcl6*^*LKO*^*Fxr*^*LKO*^) compared with their full-body *Fxr* knockout counterparts (*Bcl6*^*LKO*^*Fxr*^*KO*^) (Figure 14*C*). This reduction in *Cyp7a1* was likely due to elevated ileal *Fgf15*, which we observed in *Bcl6*^*LKO*^*Fxr*^*LKO*^ compared with both control (*Bcl6*^*fl/fl*^*Fxr*^*fl/fl*^) and *Bcl6*^*LKO*^*Fxr*^*KO*^ mice (Figure 14*D*). These data indicate that liver-intrinsic FXR signaling is dispensable for the massive BA elevation observed in *Bcl6*^*LKO*^*Fxr*^*KO*^ animals, which instead is likely dependent upon ileal FXR and FGF15 enterohepatic signaling.

## Conclusion

**Figure 15:**
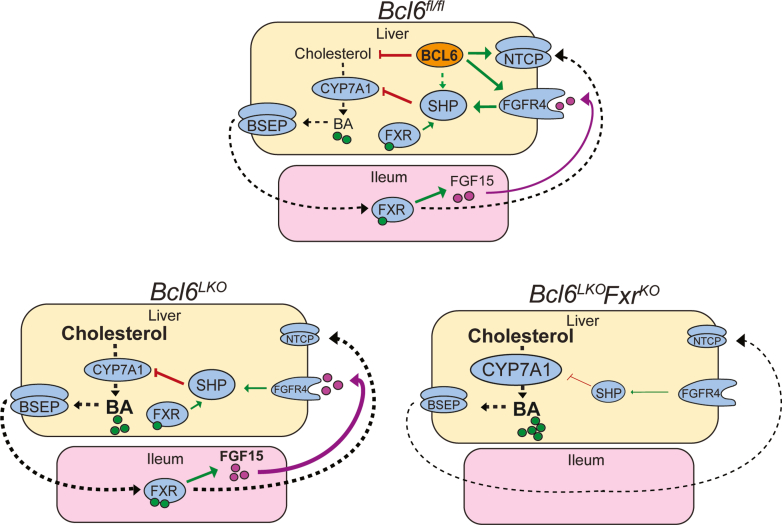
**Model for BCL6 and FXR co-regulation of BAs.** (*Top*) In control (*Bcl6*^*fl/fl*^) mice, BCL6 restrains hepatic and circulating cholesterol while enhancing NTCP and FGFR4 expression. In tandem, FXR activates SHP via enterohepatic and direct liver signaling to suppress CYP7A1 and BA production. (*Bottom left*) Loss of hepatic *Bcl6* reduces the BA transporter NTCP and FGFR4 while it increases cholesterol and CYP7A1-directed BA synthesis and circulating levels. (*Bottom right*) Combined loss of hepatic *Bcl6* and whole body *Fxr* unmasks co-dependent regulation of *Shp* by BCL6 and FXR. *Shp* levels are strongly reduced, whereas CYP7A1 is reciprocally increased. BA synthesis and levels become severely elevated, leading to cholestasis.

BAs signal through FXR to control diverse processes including lipid, glucose, and BA metabolism.^1^^,^^41^, ^42^, ^43^, ^44^ Due to their signaling and cytotoxic potential, BA synthesis must be tightly controlled to maintain homeostasis and prevent hepatic damage.^45^ FXR is well-established as a key regulator of BA levels, acting directly in the liver and indirectly in the ileum to repress hepatic BA synthesis. Although this negative feedback loop is well-established, much less is understood about the control of FXR and its transcriptional network. Our work has identified BCL6 as a suppressor of BA synthesis and levels (Figure 15, *top*). Consistent with these functions, BCL6: (1) reduces cholesterol levels; (2) activates expression of FGFR4, priming the liver for FGF15-mediated repression of *Cyp7a1*; and (3) upregulates expression of the BA reuptake transporter NTCP. These findings support a model wherein BCL6 primes the liver through multiple mechanisms to shut down BA synthesis and induce BA recycling to maintain BA homeostasis.

In the absence of *Bcl6* (Figure 15, *bottom left*), levels of the BA precursor 7a-C4, were substantially higher in *Bcl6*^*LKO*^ male mice compared with controls (Figure 4*D*), indicating that loss of hepatic *Bcl6* increases BA synthesis. Although 7a-C4 is the direct product of CYP7A1, we surprisingly found that liver levels of CYP7A1 protein were not increased by *Bcl6* ablation (Figure 6*D*). This could reflect that *Bcl6* deletion facilitates proper CYP7A1 localization or retention in the endoplasmic reticulum, enhances CYP7A1 enzymatic activity through allosteric regulation or post-translational modification, or increases CYP7A1 reaction rate by increasing its substrate levels (cholesterol) in the endoplasmic reticulum. To date, little is known about the regulation of CYP7A1 beyond its transcriptional control,^46^, ^47^, ^48^, ^49^, ^50^, ^51^, ^52^ so future work to understand how BCL6 influences CYP7A1 may illuminate new regulatory aspects of classical BA synthesis.

BA metabolism differs between sexes, with females exhibiting a larger BA pool, higher serum BA levels, reduced BA excretion, and reduced cholesterol conversion to BAs compared with males.^53^, ^54^, ^55^ Expression of the key enterohepatic hormone *Fgf15* is also reportedly higher in the ilea of females.^55^ Notably, BCL6 is sexually dimorphic with lower expression in the livers of females.^19^, ^20^, ^21^ We correspondingly found that the total BA pool, BA synthesis rate, and ratio of primary to secondary BAs were not substantially impacted in females with hepatic *Bcl6* deletion, in contrast to the marked BA changes observed in *Bcl6*^*LKO*^ males (Figure 4*A–F*; Figure 5*A–F*). We additionally found that hepatic BCL6 regulated ileal FXR signaling in a sex-biased manner, with *Bcl6*^*LKO*^ males exhibiting increased expression of *Fgf15* and FXR target genes compared with controls, an effect which was not evident in females (Figure 6*B*; Figure 7*B*). These data support a role for hepatic BCL6 as a regulator of male-biased BA metabolism in mice. Future studies to test the impact of BCL6 on intestinal BA composition, signaling, and the microbiome may further reveal functional consequences of its sex-dependent BA regulation.

We found evidence of epigenetic crosstalk between BCL6 and FXR, including co-binding along the *Shp, Klb, and Bsep* loci (Figure 1). To understand their relationship in BA metabolism, we generated a model lacking both hepatic *Bcl6* and whole body *Fxr* (*Bcl6*^*LKO*^*Fxr*^*KO*^) (Figure 15, *bottom right*). Previous work has suggested that FXR is not the sole regulator of *Shp* expression and BA synthesis; *Fxr*^*KO*^ mice retain some *Shp* expression and only show a modest increase in CYP7A1 and BA levels compared with the massive elevations observed with dual deletion of both *Fxr* and *Shp*.^56^^,^^57^ In the present study, we found that dual deletion of hepatic *Bcl6* and whole body *Fxr* (*Bcl6*^*LKO*^*Fxr*^*KO*^) results in a profound loss of hepatic SHP, in turn causing induction of CYP7A1 (particularly in males), massive BA elevation, and liver injury similar to that observed with combined deletion of *Fxr* and *Shp*.^56^ Remarkably, these effects were not observed when co-deletion of *Bcl6* and *Fxr* was restricted to hepatocytes, indicating a critical interaction between FXR enterohepatic signaling and BCL6-directed regulation of BA metabolism in the liver.

Our findings reveal BCL6 as an FXR co-regulatory transcription factor which, in the absence of whole body *Fxr*, is critical for maintaining *Shp* expression and protecting the liver from cholestatic liver damage. This co-regulatory role of BCL6 could have clinical implications. For instance, cholestatic liver diseases are sometimes associated with reduced *Fxr* expression or activity,^58^, ^59^, ^60^ and specific forms of cholestasis are associated with loss-of-function mutations in FXR.^61^, ^62^, ^63^ FXR agonists are a major therapy for cholestatic diseases, although ∼30% to 40% of patients with primary biliary cholangitis (PBC) do not respond to ursodeoxycholic acid (UDCA),^64^ and high doses of obeticholic acid (OCA) have been linked with worsening cholestasis in patients with PBC and advanced cirrhosis.^65^ Thus, our results raise the possibility that interventions to raise hepatic BCL6 could be therapeutic for cholestatic disease.

## Materials and Methods

### Mice

*Bcl6*^*fl/fl*^ mice, which contain loxP sites between exons 5 and 6 of the mouse *Bcl6* locus, were generated by the UC David Mouse Biology Program.^66^ *Bcl6*^*fl/fl*^ mice were crossed with *Albumin-Cre* (Jackson Laboratories, Stock #003574) to generate *Bcl6*^*LKO*^ mice. *Fxr*^*KO*^ (Stock #004144) and *Fxr*^*fl/fl*^ mice (Stock #028393) were acquired from Jackson Laboratories. Mice were housed with a 14:10 light:dark cycle. All experiments were performed in ad libitum-fed or 4-hour fasted animals unless otherwise stated. All animal care and procedures were conducted in accordance with regulations of the Institutional Animal Care and Use Committee at Northwestern University, protocol IS000020416. FGF19 treatment: Ad libitum-fed *Bcl6*^*fl/fl*^ and *Bcl6*^*LKO*^ males were treated with intraperitoneal (i.p.) injection of 0.1 mg/kg rhFGF19 (Peprotech) dissolved in phosphate buffered saline (PBS) or corresponding PBS vehicle. Mice were fasted 3 hours post-injection, and then livers were harvested for RNA and protein. AAV-TBG-SHP injections: Liver-specific (AAV8) adeno-associated virus driven by the thyroid binding globulin promoter (TBG) containing the *Nr0b2* cDNA (AAV-TBG-SHP) or GFP (AAV-TBG-GFP) were acquired from Vector Biolabs. *Bcl6*^*LKO*^*Fxr*^*KO*^ and *Fxr*^*KO*^ mice were retro-orbitally injected with 1.5 × 10^11^ GC of either AAV-TBG-SHP or AAV-TBG-GFP and harvested 4 weeks later for analysis.

### Serum/Liver Lipid Quantification

Liver cholesterol was isolated using the Folch method, in which tissues were homogenized in 1 mL of methanol and then incubated overnight in a 1:2 methanol:chloroform solution. 0.9% NaCl was added to homogenates, which were then incubated overnight. The subsequent chloroform layer was extracted and dried under nitrogen gas. The lipids were resuspended in 2-propanol prior to quantification. Serum and liver triglycerides and cholesterol levels were quantified using the Infinity Thermo Fisher kit for triglycerides or cholesterol. Lipoprotein separation of serum was performed using fast protein liquid chromatography (FPLC) at the Vanderbilt Mouse Metabolomic Phenotyping Center (MMPC).

### qPCR

∼25 mg of livers or terminal ilea (last 2 cm of ileum just proximal to cecum) were collected in RNALater (ThermoFisher). Samples were bead-homogenized in Trizol (ThermoFisher). 20% chloroform by volume was added, and the aqueous phase was isolated following centrifugation. RNA was further purified using the RNAeasy kit (Qiagen). 1 ug RNA was converted to cDNA using the iScript cDNA synthesis kit (BioRad). qPCR was performed using iTaq Universal SYBR Green Supermix (BioRad). All gene expression was quantified using the standard curve method and normalized to the housekeeping gene *36b4*. See Table 2 for primer sequences.

**Table 2:** Primer Sequences

Name	Forward Primer	Reverse Primer
*Dhcr24*	CGCCTGTCACTTGGAACATTAG	CCTAGCTACCACCTGGATCATT
*Dhcr7*	AGAGCTGAATTCACACGGATAC	CTCCAAGCAGAGAGACATGAAA
*Lss*	GTGTCTTGGCTGGGTGATAA	GACACCAACACTGACCCTATC
*Hmgcr*	CCAGAAGCTTTCGTCAGTAGAG	CTCTGCTTGTAGTCTCTGCTT
*Mvk*	GGTGGCCTTGAACTTGAGAA	CATCCCAGACCTGCTTAATACC
*Sqle*	AGAGCCCGACAGGATAGTT	GATGGGCATTGAGACCTTCTAC
*Ldlr*	ATCCACCGCAACATCTACTG	GGAACAGTGTCCTCCTCTTTAC
*Lcat*	GAAAGAGGAGCAGCGCATAA	GTCTTGGACGGTGTAGTTGAAG
*Vldlr*	GTGACCACAGCAGTATCAGAAG	CTGCCATCACTAAGAGCAAGAG
*Abcg1*	ACCCGCCTGTCATGTTCTTT	CACCACTTGGAAGCAGGAGG
*Apoe*	TGAACCGCTTCTGGGATTAC	CATCAGTGCCGTCAGTTCTT
*Cyp2c54*	TTAAAGGAGCCCAGGAAGATG	TTGACTCTGTCCCACCAATAAA
*Cyp2c67*	CAAGAGGAAGCACAGTGGCTCA	GGAAAACAATGGAGCAGATGACAT
*Cyp4v3*	CACCTTGGAAGACCTGAAGAAA	GCTAAGACTCCGGGCAAATAA
*Cyp3a11*	CCGAGTGGATTTTCTTCAGC	GAGCCTCATCGATCTCATCC
*Cyp2c70*	GATTGACCAGGGAGATGAGTTT	CGGGTTTGTTTCCATGTTTCTC
*Cyp2a12*	TGCTCCTCCTAGCCATTCTG	CAGGGCCATAGTGCTCTTGG
*Cyp2a22*	GGCACTGATGTGTTCCCTATAA	TCTTCAACTGTCCCTTGTCATC
*Cyp2b9*	CTCCACTATGGAGTCCTGCTCA	GCGGTCATCAAGAGTTGGTAGC
*Cyp3a59*	GCTATGATGCCACAAGCACTTCC	TCAGGGCATCATAGGTGACAGG
*Cyp17a1*	GGATGCACAGGTTGAGGTTAG	GGATAGGAGTGAGGAGGATTGT
*Fxr (Nr1h4)*	ATCCCAGATCTCACAGAGGAG	TCCGGACATTCAACCATCAC
*Shp (Nr0b2)*	CGATCCTCTTCAACCCAGATG	AGGGCTCCAAGACTTCACACA
*Fgf15*	GATATACGGGCTGATTCGCTAC	AGATGGTGCTTCATGGATCTG
*Osta (Slc51a)*	CTTCTGTCCCTCAGCCTTATC	GTTCAACCCACTGCACTTTAC
*Ostb (Slc51b)*	CCTGCATCTTGATGACTCCATA	TTCTCTTTCAACTCAGGTTCCC
*Ibabp (Fabp6)*	TATGAGCGCGTAAGCAAGAG	CTAGCAGTGGTGATCCATGAAA
*Bcl6*	TGCAGATGGAGCATGTTG	ACCTCGGTAGGCCATGAT
*Fgfr4*	CCTGAGGCCAGATACACAGATA	GGATGACTTGCCGATGATACAC
*Klb*	CCAAGTCAGCTGTTCCTCTATG	CATCTGTCTTCCAACTCCCTTC
*Cyp7a1*	AGCCAGAGTCCAATGCTTAG	CTCATCTCACACCAGGGTAAAT
*Cyp8b1*	TTTCTGAGGGAGCAAGGAATAG	GGAATAAGAGGACCCAGAAACA
*Cyp27a1*	TTGCCTTGGAAGCCATCAC	GGCAGCCAATCCTTTTCTCA
*Cyp7b1*	CGAGAAGTGCAGGAGGATATG	GTGTATGAGTGGAGGAAAGAGG
*Ntcp (Slc10a1)*	TCTCTGCTCTCTTCCGACTAA	GGTGACATTGAGGATGGTAGAA
*Bsep (Abcb11)*	GCTCATCGCTTGTCTACTATCC	CCTTCTGGTCCATCAGTTTCTT
*Col1a1*	AGACCTGTGTGTTCCCTACT	GAATCCATCGGTCATGCTCTC
*Tnfa*	ACTCCCAGGTTCTCTTCAAGG	GTGGGTGAGGAGCACGTAGT
*Tgfb*	CTCCCGTGGCTTCTAGTGC	GCCTTAGTTTGGACAGGATCT
*Lox*	CCTGGCCAGTTCAGCATATAG	GTAAGAAGTCCGATGTCCCTTG
*Cd4*	TCCTAGCTGTCACTCAAGGGA	TCAGAGAACTTCCAGGTGAAGA
*Cd8*	CCGTTGACCCGCTTTCTGT	CGGCGTCCATTTTCTTTGGAA
*F480*	TTGTACGTGCAACTCAGGACT	GATCCCAGAGTGTTGATGCAA
*aSma*	GTCCCAGACATCAGGGAGTAA	TCGGATACTTCAGCGTCAGGA
*Gfp*	AGTCCGCCCTGAGCAAAGA	TCCAGCAGGACCATGTGATC
*36b4*	AGATGCAGCAGATCCGCA	GTTCTTGCCCATCAGCACC
*Shp(Nr0b2)_Promoter*	GCATGGAAATGGGCATCAATAG	TCGGATGACTCAAGTGCATAAA
*Shp(Nr0b2)_Downstream*	CAGTTGTCTACAGGGCTTTCA	CCGGTGAGAAGGATCCAAAC

### Western Blotting

Fifty mg liver samples were dounced in RIPA buffer and centrifuged to isolate the liquid fraction. Bicinchoninic acid (BCA) assay (ThermoFisher) was used to quantify protein. Lysates were denatured by boiling for 5 minutes in loading buffer. Twenty to 40 ug of lysates were loaded into pre-cast gels (BioRad) and transferred to 0.4 um polyvinylidene fluoride (PVDF) membranes (Immobilon). Membranes were blocked in 5% milk in TBST or PBST. Primary antibodies for BCL6 (sc-7388, Santa Cruz) at 1:200, FXR (E4B8P, Cell Signaling) at 1:1000, FGFR4 (D3B12, Cell Signaling) at 1:1000, NTCP (ab131084, Abcam) at 1:1000, pERK1/2 (#9101, Cell Signaling) at 1:1000, ERK1/2 (#137F5, Cell Signaling) at 1:1000, CYP7A1 (18054-1-AP, ProteinTech) at 1:1000, GFP (#G10362, Invitrogen) at 1:1000, SHP (PA5-102494, Thermo Fisher) at 1:1000, CYP8B1 (Abcam, ab191910), or COL1A1 (Cell Signaling, #E8I9Z) were added and incubated overnight at 4 degrees. Secondary antibodies (Jackson ImmunoResearch) were added for 1.5 hours. All blots were imaged and quantified using the Licor Odyssey FC and ImageStudio (Licor). All protein levels were normalized to Licor Total Protein Stain.

### ChIP

Livers were harvested and dual-crosslinked by homogenizing in 2 mM disuccinimidyl glutarate for 30 minutes followed by adding 1% formaldehyde for 10 minutes. Samples were quenched in 125 mM glycine, then rinsed with PBS. Crosslinked nuclei were lysed in cell lysis buffer (0.75M NaCl, 1% Triton X, 0.5 mM Tris, .05 mM EDTA, and 0.5% NP-40) and lysed in sodium dodecyl sulfate (SDS) lysis buffer (1% SDS, 10 mM EDTA, and 50 mM Tris) using a Diagenode Bioruptor. Samples were then incubated in 5 ug of FXR antibody (E4B8P, Cell Signaling) or 2 ug of H3K27ac antibody (39133, Active Motif) overnight, and immune complexes were isolated using anti-IgG paramagnetic beads (ThermoFisher). De-crosslinked samples were further processed using the MinElute PCR purification kit (Qiagen). For H3K27ac ChIP-qPCR, DNA was diluted 1:20 and assessed via qPCR and expressed as percent of input chromatin. See Table 2 for primer sequences.

### ChIP-seq

Samples were made into libraries using the KAPA Hyperprep Kit (Kapa Biosystems) and sequenced using the Illumina NextSeq 2000 with 100 bp single-end reads. The resulting FASTQ files were aligned to the mm10 genome using Bowtie with parameters to output uniquely mapped reads.^67^ ChIP-seq samples were analyzed with HOMER.^68^ The ‘makeTagDirectory’ command with ‘-tbp1’ was used to make tag directories. Homer’s ‘-makeUCSCfile’ and ‘-bedGraphToBigWig’ commands were utilized to make BigWig files for visualization on the UCSC genome browser.

### RNA-seq

RNA from liver and ileum was purified using the RNAeasy kit (Qiagen). Sequencing libraries were made using the TruSeq Stranded mRNA Kit (Illumina), and sequencing was performed on the Illumina NextSeq 2000 using 100 bp single-end reads. Sequences were aligned to mm10 using STAR version 2.5.2 with ‘--outFilterMultimapNmax 1’ and ‘--outFilterMismatchNmax 4’.^69^ Gene expression was normalized and reads per kilobase of transcript per million mapped reads (RPKM) was quantified using HOMER.^68^ Differential expression was calculated using DEseq2.^70^ Venn diagrams were generated in R using BioVenn,^71^ and Gene Ontology analysis was performed using EnrichR.^72^

### Integrated Analysis of Motif Activity and Gene Expression Analysis

Liver RNA-seq and H3K27ac datasets from male ad libitum-fed *Bcl6*^*fl/fl*^ and *Bcl6*^*LKO*^ livers were taken from our previously published data (GSE118789). These datasets were aligned, and tag directories were generated as described above. For the RNA-seq datasets, HOMER’s ‘analyzeRepeats.pl’ with parameters ‘-noadj’, ‘-count exons’ and ‘-condenseGenes’ was used to create a raw counts file containing tags from all replicates. For H3K27ac datasets, Homer’s ‘findPeaks.pl’ with style ‘-histone’ was used to generate a list of peaks for each replicate. Homer’s ‘mergePeaks’ with ‘-d given’ was then utilized to make a merged list of all individual sample peaks. Then, ‘annotatePeaks.pl’ was used to annotate the merged list with tags from each replicate. These gene and regulatory region lists served as inputs for Integrated Analysis of Motif Activity and Gene Expression (IMAGE) to predict transcription factors driving changes in gene expression. (https://github.com/JesperGrud/IMAGE/).

### BA Quantification

Individual serum BAs were analyzed in overnight-fasted serum by the Duke Metabolomics/Proteomics Core. Total serum BAs were quantified using the Crystal Chem Mouse Serum Bile Acid kit. For liver BAs, ∼50 mg of left-lobe liver was weighed and dounced in 1 mL of 75% ethanol. Samples were transferred into glass test tubes and incubated at 50° for 2 hours. Samples were then centrifuged, transferred into vials, and stored at −20° until BAs were quantified. To measure the total BA pool, the liver, gallbladder, and small intestines were weighed and transferred to a tube containing 20 mL of 75% ethanol. Tissues were minced and homogenized for 30 seconds using a Polytron. The homogenized contents were then incubated at 50° for 2 hours and centrifuged. The resulting lysate was collected and diluted 1:4 with PBS prior to quantification. Fecal BAs were processed as previously described.^73^ For collection, mice were single-housed for 12 hours, and all feces were collected into a glass culture tube. Fecal samples were placed in a 37° incubator until completely dry and crushed with a mortar and pestle. Two mL of 75% ethanol was added to 100 mg of feces, then samples were incubated at 50° for 2 hours. Samples were centrifuged and then transferred to vials to be quantified. Liver, fecal, and total pool BA extracts were quantified using the Crystal Chem Mouse Serum Bile Acid kit per the manufacturer’s instructions.

### Serum 7a-C4, ALT, and Bilirubin Quantification

Serum 7a-C4 was quantified in overnight-fasted serum by Mayo Clinic’s Immunochemical Core Lab. Serum ALT and bilirubin were quantified by Northwestern’s Microsurgery Core using the Mammalian Liver Profile kit (Abaxis).

### Histology

One hundred mg of liver from the left lobe was fixed in 10% formalin overnight, stored in 70% ethanol, paraffin-embedded, and sectioned. Embedded samples were stained using hematoxylin and eosin (H&E), Picrosirius red, and alpha-smooth muscle actin (ThermoFisher 53-9760-82) and F4/80 (Cell Signaling #70076S) antibodies by Northwestern University’s Mouse Histology and Phenotyping Laboratory.

### Statistical Analysis

GraphPad Prism (Dotmatics) was utilized to perform statistical analyses with Student’s *t*-tests or analysis of variance (ANOVA) with Holm-Sidak’s post-hoc testing as detailed in the figure legends.

## CRediT Authorship Contributions

Ellen Fruzyna (Conceptualization: Lead; Formal analysis: Lead; Funding acquisition: Supporting; Investigation: Lead; Writing – original draft: Lead; Writing – review & editing: Supporting)

Meredith A. Sommars (Conceptualization: Supporting; Formal analysis: Supporting; Funding acquisition: Supporting; Investigation: Supporting)

Yasu Omura (Investigation: Supporting)

Kristine M. Yarnoff (Investigation: Supporting)

Janice C. Wang (Investigation: Supporting)

Christopher R. Futtner (Investigation: Supporting)

Richard M. Green (Conceptualization: Supporting)

Grant D. Barish (Conceptualization: Lead; Funding acquisition: Lead; Supervision: Lead; Writing – original draft: Lead; Writing – review & editing: Lead)
